# An odorant receptor from *Anopheles gambiae* that demonstrates enantioselectivity to the plant volatile, linalool

**DOI:** 10.1371/journal.pone.0225637

**Published:** 2019-11-21

**Authors:** Robert Mark Huff, R. Jason Pitts

**Affiliations:** Department of Biology, Baylor University, Waco, Texas, United States of America; University of Richmond, UNITED STATES

## Abstract

Insects express chemical receptors within sensory neurons that are activated by specific cues in the environment, thereby influencing the acquisition of critical resources. A significant gap in our current understanding of insect chemical ecology is defining the molecular mechanisms that underlie sensitivity to plant-emitted volatiles. Linalool is a commonly-occurring monoterpene that has various effects on insect behavior, either acting as an attractant or a repellent, and existing in nature as one of two possible stereoisomers, (*R*)-(–)-linalool and (*S*)-(+)-linalool. In this study, we have used a cell-based functional assay to identify linalool and structurally-related compounds as ligands of Odorant receptor 29, a labellum-expressed receptor in the malaria vector mosquito, *Anopheles gambiae* (AgamOr29). While (*R*)-(–)-linalool activates AgamOr29, a mixture of the (*R*) and (*S*) stereoisomers activates the receptor with higher potency, implying enantiomeric selectivity. Orthologs of Or29 are present in the genomes of Anophelines within the Cellia subgenus. The conservation of this receptor across Anopheline lineages suggests that this ecologically important compound might serve as an attraction cue for nectar-seeking mosquitoes. Moreover, the characterization of a mosquito terpene receptor could serve as a foundation for future ligand-receptor studies of plant volatiles and for the discovery of compounds that can be integrated into push-pull vector control strategies.

## Introduction

Mosquitoes use sensory receptors to detect environmental chemical cues that influence resource acquisition success. Among these are the characteristically female behaviors of animal host seeking [1,2] and oviposition site selection [3,4], plus the female and male behaviors of nectar locating [5] and resting site selection [6]. Chemosensory receptor proteins are expressed in sensory neurons and comprise three major families in insects: odorant receptors (Ors) [7], gustatory receptors (Grs) [8], and variant ionotropic glutamate receptors (Irs) [9]. The interactions between chemoreceptors and their environmental ligands, while studied extensively in some insects, remain uncharacterized for the vast majority of mosquito species and are therefore of interest for further scientific inquiry [7]. Closing the gaps in our understanding of the molecular detection of ecologically relevant odor compounds will allow us to determine how receptor-ligand pairs can influence mosquito behaviors. Past studies have helped define important chemical cues that attract mosquitoes to suitable animal hosts and oviposition sites [10–13], as well as nectar sources [14–16]. However, more studies are needed to elucidate the molecular mechanisms that determine how mosquitoes are able to locate suitable nectar sources, especially the activation of chemoreceptors by plant produced volatile organic compounds (VOCs).

Linalool is a commonly-occurring plant VOC that is emitted by flowers and exists as one of two stereoisomers, (*R*)-(–)-linalool and (*S*)-(+)-linalool. Linalool is sensed by a wide range of animal species and is known to affect the behavior of insects, especially Lepidoptera [17,18]. For example, linalool is emitted by *Clarkia breweri* Grey [Green] flowers and attracts moth pollinators [17] and activates receptor neurons of noctuid adults [19–21]. In the cabbage moth, *Mamestra brassicae*, sensory neurons respond more strongly to (*R*)-(–)-linalool [22], while in the sphinx moth, *Manduca sexta*, (*S*)-(+)-linalool elicits increased oviposition, indicating that enantiomeric selectivity may be an important aspect of insect chemical ecology [23]. In mosquitoes, linalool has been shown to evoke different valencies, depending on the species and context of presentation. For example, linalool can act a spatial repellent to host seeking in *Aedes aegypti* [24] and has been shown to attract *Culex pipiens pallens* [25] in an Y-olfactometer both as a unitary compound as a component of blends. Importantly, the molecular mechanism(s) of these modes of action are unknown, especially in the context of potential differential responses to the stereoisomers. Previous work has demonstrated enantiomeric selectivity toward environmental ligands in mosquitoes. For example, orthologs of the *Or8* receptor in the vector species, *A*. *aegypti*, as well as the strict nectar feeding mosquito, *Toxorhynchites amboinensis*, are selective for the (*R*)-(–)-enantiomer of the volatile organic 1-octen-3-ol [26, 27].

Interestingly, humans are also able to distinguish linalool isomers, with the former having the smell of lavender and the latter the smell of coriander [28]. Moreover, linalool elicits multiple effects on mammalian and insect nervous systems, variously acting as an anxiolytic, anesthetic, or neurotoxic compound [29]. While the modes of action of linalool on neurons and additional cell types remain to be investigated, the number of possible receptor targets is quite varied and includes serotonin, GABA, NMDA, and nicotinic acetylcholine receptors, among others [30–36].

The significance of linalool as an environmental odor cue in Anophelines is an open question, but the availability of multiple genomes has facilitated an initial investigation into its potential mechanisms of action [37]. In this study, we have used a cell-based functional assay to screen a small library of compounds from which we identified linalool as an activator of *Anopheles gambiae* Or29 (AgamOr29). Both (*R*)-(–)-linalool and a mixture of stereoisomers activate AgamOr29 in a concentration-dependent manner. However, (*R*) and (*S*) in combination activate the receptor with higher potency than the (*R*) isomer alone, implying that the (*S*)-(+)-linalool enantiomer is likely to be the more effective ligand and that Or29 is an enantioselective receptor. The conservation of the Or29 receptor across Anophelines and its expression in the head appendages of *A*. *gambiae*, especially the labellar lobes, suggests that this ecologically important compound might serve as an attraction cue for nectar-seeking mosquitoes. Moreover, this receptor could serve as a target for the development of new excito-repellents that are specific to malaria vectors.

## Materials and methods

### Phylogenetic analysis

*A*. *gambiae* Or29 homologs were identified in all available Anopheline genomes [37] on NCBI via tBLASTn or BLASTp searches. Geneious Prime^2019^ software (Biomatters Limited, USA) was utilized for multiple sequence alignment as well as phylogenetic tree construction using the Neighbor-Joining method with 1000 bootstrap pseudoreplicates.

### Transcriptional analysis

Transcriptional analysis of mosquito sensory appendages and bodies were derived from previously published studies [38–40]. Normalized transcript abundance values were reported as were available in the literature as either FPKM (Fragments per Kilobase per Million Reads) or RPKM (Reads per Kilobase per Million Reads).

### Gene cloning and sequencing

AgamOrco and AgamOr29 templates were provided by the laboratory of Dr. Laurence Zwiebel (Vanderbilt University). Coding regions were cloned into the pENTR^*TM*^ vector using the Gateway^*R*^ directional cloning system (Invitrogen Corp., Carlsbad, CA, USA) and subcloned into the *Xenopus laevis* expression destination vector pSP64t-RFA. Plasmids were purified using GeneJET Plasmid Miniprep Kit (ThermoFisher Scientific, Waltham, MA, USA) and sequenced in both directions to confirm complete coding regions.

### Chemical reagents

The chemicals (Supplementary Table 1) used for the deorphanization of AgamOr29 were obtained from Acros Organics (Morris, NJ, USA), Alfa Aesar (Ward Hill, MA, USA), and Thermo Fisher Scientific (Waltham, MA, USA) at the highest purity available (S1 Table). AgamOr29 chemical class responsiveness was determined by using complex odorant blends comprising 72 unique chemical compounds. Odorants were made into 1M stocks in 100% DMSO. Compounds in blends were grouped by chemical class and were combined and diluted to 10^−4^ M in ND96 perfusion buffer (96mM NaCl, 2mM KCl, 5mM MgCl_2_, 0.8mM CaCl_2_, and 5mM HEPES).

**Table 1:** Odorant blends used in AgamOr29/Orco two-electrode voltage clamp recordings.

Indoles	Ketones	Lactones	COOHs	Terpenes	Alc/Ald 1	Alc/Ald 2
2,3-dimethylindole	(+/–)-camphor	alpha-angelicalactone	acetic acid	(–)-alpha-pinene	2-propanol	1,8-cineole
2-methylindole	4-ketoisophorone	delta-dodecanolactone	decanoic Acid	(–)-limonene	4-tert-butylphenol	1-hexanol
3-methylindole	6-methyl-5-hepten-2-one	delta-nonanolactone	glycine	(+)-nootkatone	DL-menthol	1-octen-3-ol
indole	acetone	delta-octanolactone	heptanoic acid	(1S)-(–)-beta-pinene	ethanol	anisole
indole-3-carboxyaldehyde	cis-jasmone	delta-valerolactone	hexanoic acid	alpha terpineol	farnesol	cis-2-hexen-1-ol
methyl-indole-3-carboxylate	coumarin	gamma-octanolactone	isovaleric acid	citral	glycerol	cyclohexanol
methyl salicylate	cyclopentanone	gamma-undecalactone	(L)-(+)-lactic acid	citronellol	methanol	hexanal
	ethyl acetate	gamma-valerolactone	linalyl acetate	geranyl acetate	n-butanol	isoeugenol
	geranyl acetone		linoleic acid	N,N-diethyl-m-toluamide	o-cresol	(*R*)-(–)-linalool
	isophorone		n-nonanoic acid	trans-cinamaldehyde	p-cresol	trans-2-hexen-1-ol
			octanoic acid			trans-2-hexenal

Composition of the odorant blends used during the deorphanization of AgamOr29. Compounds were grouped by chemical classes and mixed at equimolar concentrations of [10^−4^ M] each.

### Two-electrode voltage clamp of *Xenopus laevis* oocytes expressing AgamOrco and AgamOr29

AgamOrco and AgamOr29 cRNA were synthesized from linearized pSP64t expression vectors using the mMESSAGE mMACHINE^®^ SP6 kit (Life Technologies). Stage V-VII *Xenopus laevis* oocytes were ordered from Xenopus1 (Dexter, MI, USA) and incubated in ND96 incubation media (96 mM NaCl, 2mM KCl, 5mM HEPES, 1.8mM CaCl_2_, 1mM MgCl_2_, pH 7.6) supplemented with 5% dialyzed horse serum, 50 μg/mL tetracycline, 100μg/mL streptomycin, 100μg/mL penicillin, and 550 μg/mL sodium pyruvate. Oocytes were injected with 27.6 nL (27.6 ng of each cRNA) of RNA using the Nanoliter 2010 injector (World Precision Instruments, Inc., Sarasota, FL, USA). Odorant-induced currents off oocytes expressing AgamOrco and AgamOr29 were recorded using the two-microelectrode voltage-clamp technique (TEVC). The OC-725C oocyte clamp (Warner Instruments, LLC, Hamden, CT, USA) maintained a -80mV holding potential. To measure the effect of the blends on AgamOr29, we used 10^−4^ M concentration blends for 10s. Current was allowed to return to baseline between drug administrations. Data acquisition and analysis were carried out with the Digidata 1550 B digitizer and pCLAMP10 software (Molecular Devices, Sunnyvale, CA, USA). A tuning curve was generated using a panel of 7 odorants including (*R*)-(–)-linalool and closely related structural odorants. All chemicals used were administered at 10^−4^ M. All data analyses were performed using GraphPad Prism 8 (GraphPad Software Inc., La Jolla, CA, USA). For the establishment of a concentration-response curves, oocytes were exposed to (*R*)-(–)-linalool or a racemic mixture of (*R*)-(–)-linalool and (*S*)-(+)-linalool (10^−7^ M to 10^−3^ M). To measure the effect of the compounds on the oocytes, odorants were perfused for up to 30s or until peak amplitude was reached. Current was allowed to return to baseline between chemical compound administrations.

## Results

### Odorant receptor 29 homologs

**Fig 1:**
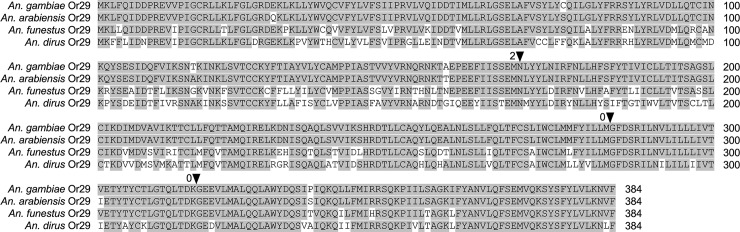
Anopheline Or29 alignment. Amino acid alignment of Or29 orthologs from representative Anopheline species. Conserved residues are highlighted in grey. Positions and phases of introns are indicated with filled triangles. Amino acid numbering is indicated at the end of each line.

**Fig 2:**
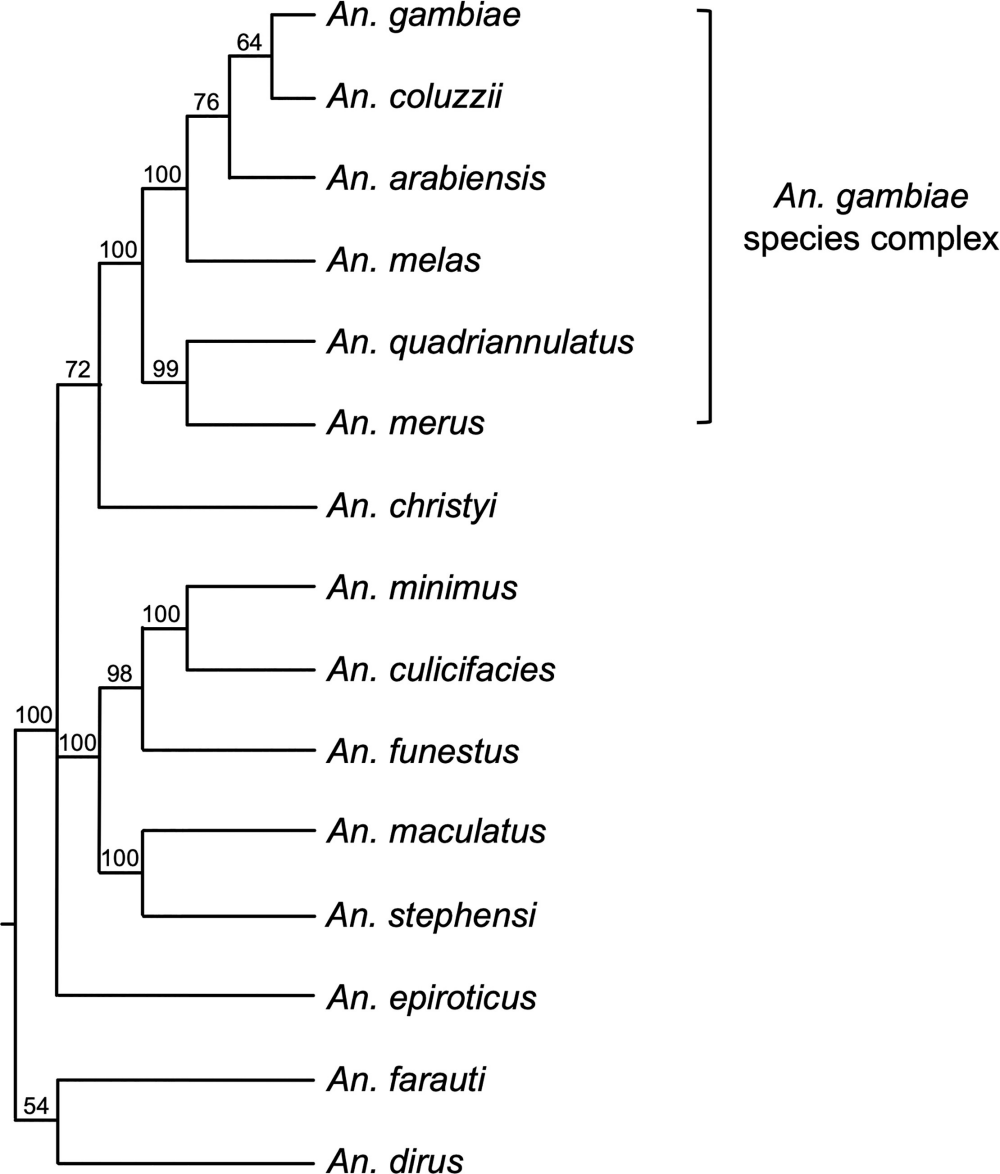
Phylogeny of Or29 homologs. Neighbor joining tree showing relationships of Anopheline Or29 peptides. Node values signify bootstrap values. Labels represent VectorBase IDs for corresponding proteins.

The *A*. *gambiae* genome encodes a single copy of Or29 (AGAP0009111), with several apparent 1:1 orthologs encoded in the genomes of additional Anopheline species identified via BLAST searches and reannotated to correct for conserved intron positions (S1 and S2 files). As shown in Fig 1 for four representative amino acid sequences, the conceptual translations of Anopheline Or29 orthologs are very similar in length (384 aa) and have pairwise identities ranging from 70% to 99% (average identity 80%), with conserved residues spanning their entire lengths (S2 Table; S1 Fig). A phylogenetic tree was constructed using the Or29 conceptual translations from 15 species with high bootstrap support for most branches (Fig 2). The Or29 relationships conform to previously described phylogenies of the Anophelines [37, 41, 42], with Or29 orthologs encoded in the genomes of species in the *A*. *gambiae* complex appearing as a single clade with 100% bootstrap support (Fig 2). Interestingly, Or29 orthologs were only identified in species within the Cellia subgenus and could not be identified in species from other subgenera for which genomes are available, i.e. Anopheles or Nyssorhynchus [41, 42]. Another odorant receptor encoded in *A*. *gambiae*, Or53 (AGAP009390), is also highly homologous to Or29, having 60% amino acid identity (S2 Fig) [43]. Anophelines in other subgenera encode at least one Or53 paralog with strictly conserved intron positions correlating to the introns Or29, suggesting that Or53 is the likely ancestral gene, with Or29 being gained via duplication in the Cellia linages [42].

### AgamOr29 expression in chemosensory appendages

**Fig 3:**
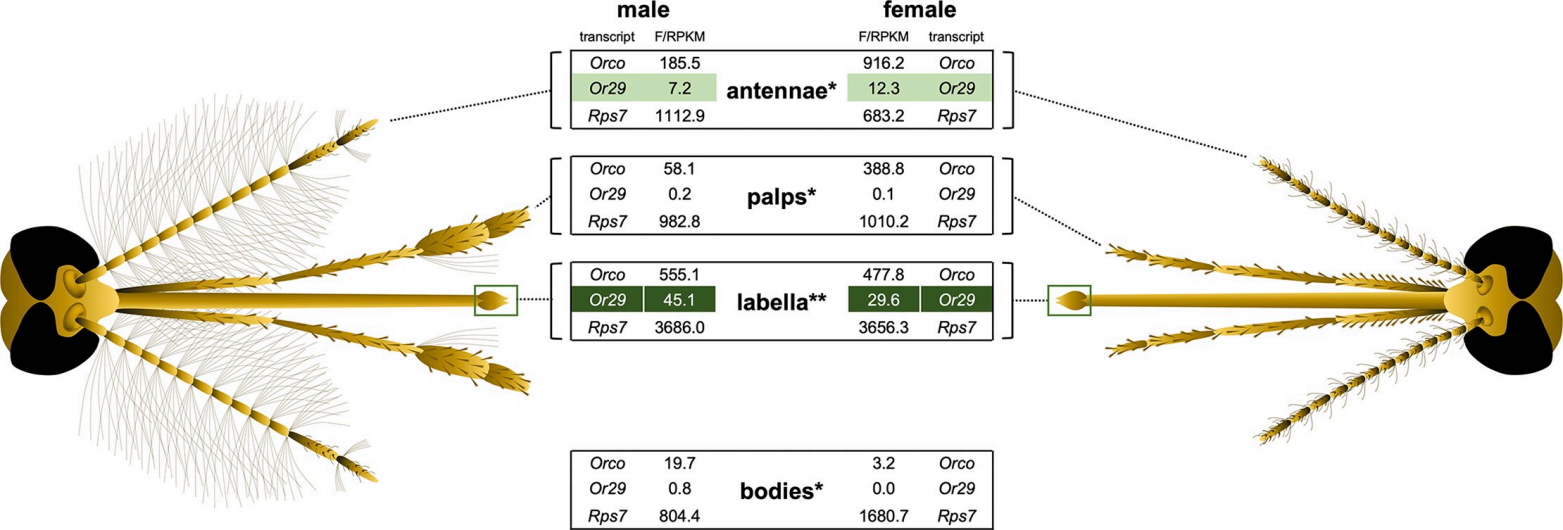
AgamOr29 expression in chemosensory appendages. Expression values in Reads Per Kilobase per Million (RPKM) or Fragments Per Kilobase per Million (FPKM) for the AgamOr29 transcript (AGAP009111) in male or female *A*. *gambiae* head appendages and whole bodies. Data from *Pitts et al. 2011 or **Saveer et al. 2018.

To better understand the expression profile of AgamOr29, we sought to determine the spatial expression of the transcript within sensory appendages of adult mosquitoes by analyzing published RNA sequencing data sets [38–40]. We have summarized available expressional data for the antennae, maxillary palps, labella, and whole bodies (Fig 3). AgamOr29 is expressed at high levels in the labella of adult mosquitoes of both sexes and is also present at lower levels in the antennae of both sexes (Fig 3). Very low expression has been observed in the maxillary palps and whole bodies (minus sensory appendages) and may be considered background (Fig 3). Or29 is also expressed in the antennae of *Anopheles quadriannulatus* females, but we were unable to find additional information about the expression of Or29 orthologs in other species [37].

### AgamOr29 is tuned to linalool

**Fig 4:**
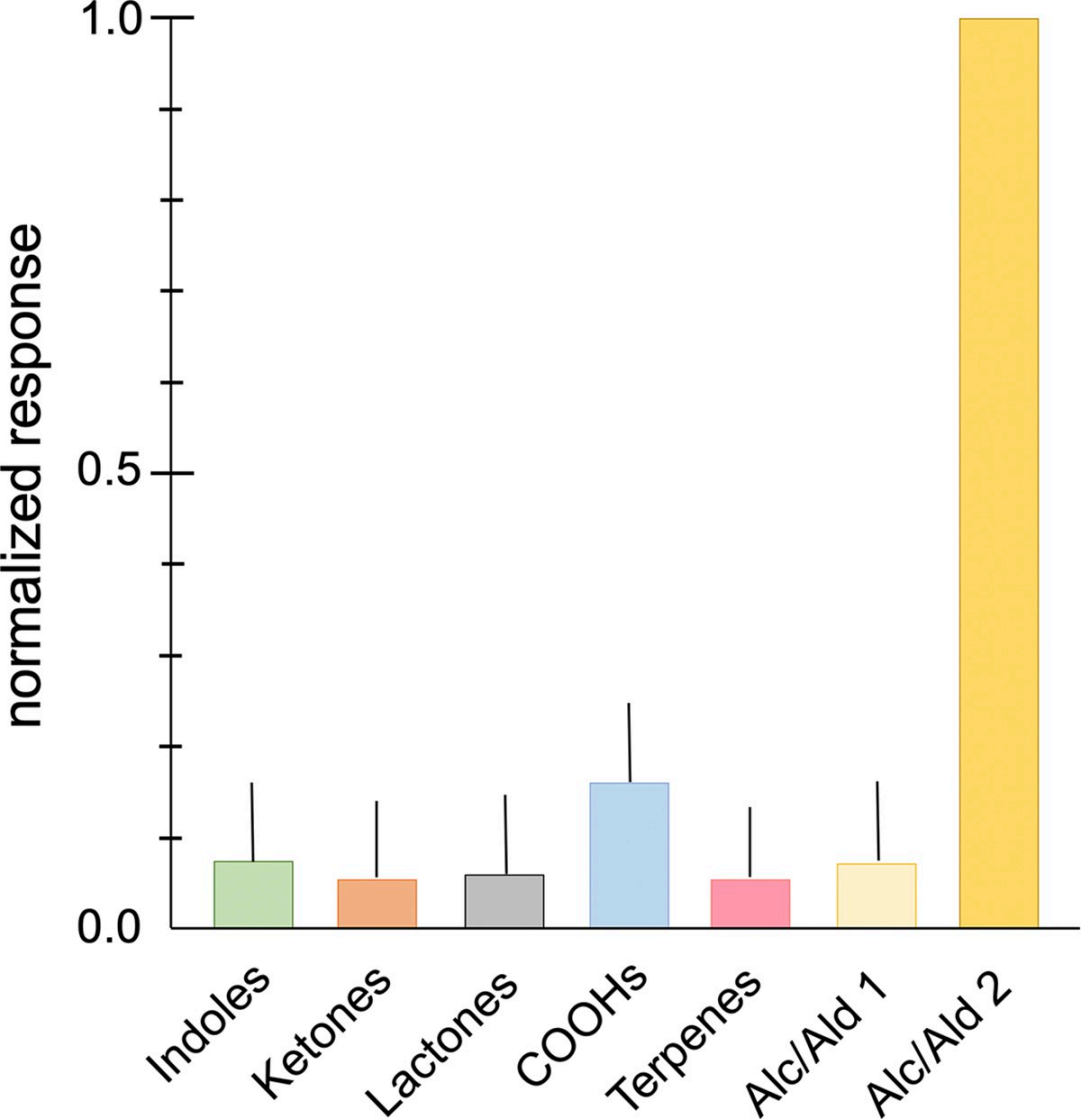
AgamOr29 Deorphanization. Normalized mean current responses of oocytes (n = 10) co-injected with AgamOr29+AgamOrco to odorant blends [10^−4^ M] grouped by chemical classes. Standard errors are indicated in the positive direction only.

**Fig 5:**
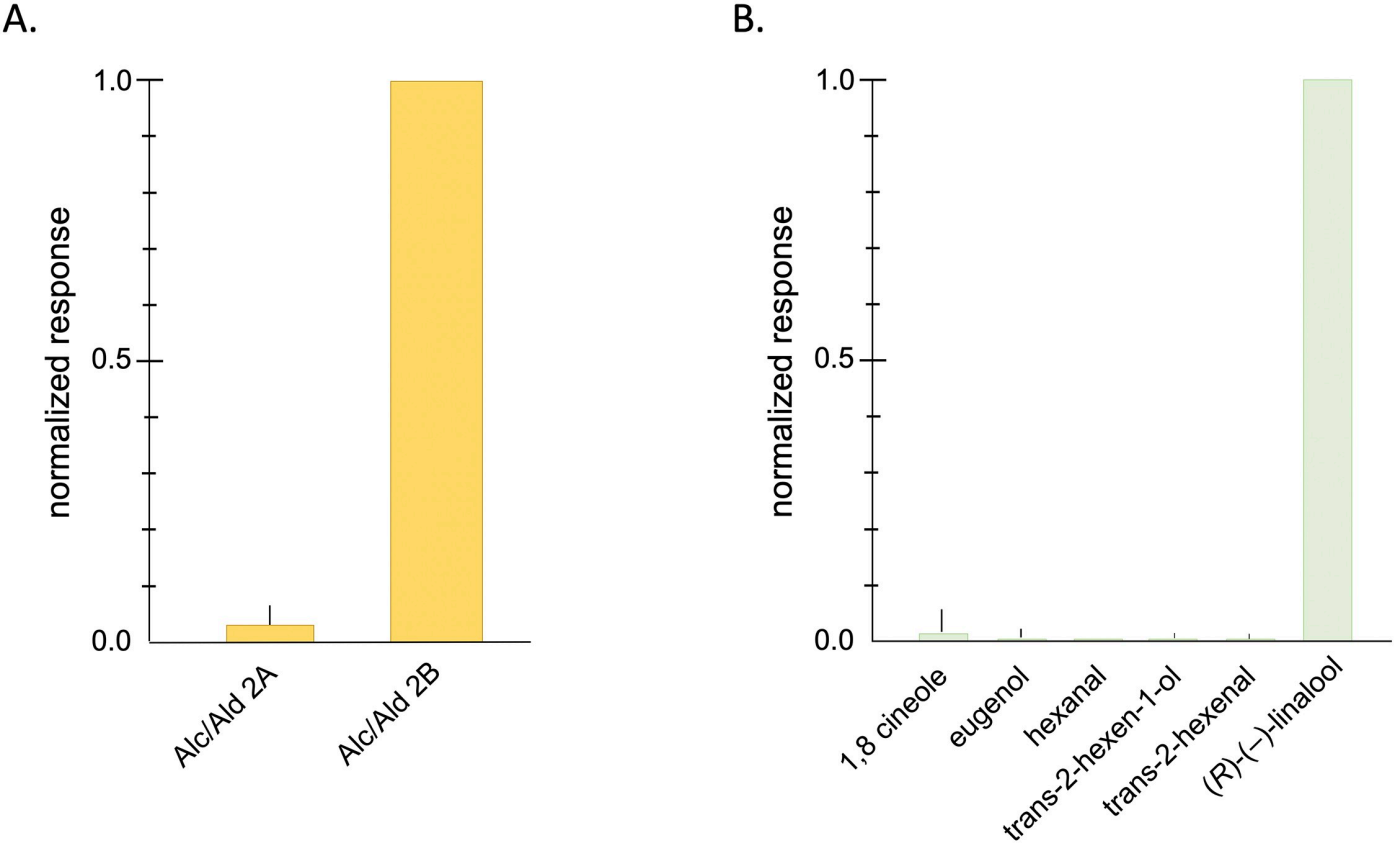
AgamOr29 alcohol response profile. (A) Alcohol/Aldehyde blend 2 was further split into two blends, Alc/Ald 2A and Alc/Ald 2B. Normalized mean current responses of oocytes (n = 10) coinjected with AgamOr29 + AgamOrco to blends 2A and 2B [10^−4^ M]. Standard errors are indicated in the positive direction only. (B) Normalized mean current responses of oocytes (n = 10) coinjected with AgamOr29 + AgamOrco to individual compounds comprising Alcohol/Aldehyde blend 2B [10^−4^ M]. Standard errors are indicated in the positive direction only.

In order to investigate the responsiveness of AgamOr29 to odorants, we expressed AgamOr29 in combination with the coreceptor, AgamOrco, in *Xenopus laevis* oocytes and recorded inward current responses of the receptor complex to multiple odorant blends using the two-electrode voltage clamp technique. The odorant blends used were grouped into six chemical classes based on structure and comprised a total of 72 distinct chemical compounds (Table 1). Compounds in odorant blends were each delivered at 10^−4^ M concentrations and applied via buffered perfusion. Oocytes expressing the AgamOr29/Orco receptor complex were most strongly activated by Alcohol/Aldehyde blend 2, with at least 7-fold greater amplitude responses on average than any other odorant compound blend (Fig 4). Oocytes expressing either the AgamOr29 or AgamOrco subunits alone were unresponsive to any of the odorant blends (S3 Table). We next split the Alcohol/Aldehyde 2 blend in into two different blends each containing 6 of the original twelve compounds (Alc/Ald 2A and 2B). Alcohol blend 2B elicited strong responses from oocytes expressing AgamOr29 and AgamOrco (Fig 5A). Of the individual alcohols present in alcohol blend 2B, (*R*)-(–)-linalool evoked large amplitude responses while the remaining alcohol compounds showed only baseline levels (Fig 5B).

### AgamOr29 responds to stereoisomers of linalool

**Fig 6:**
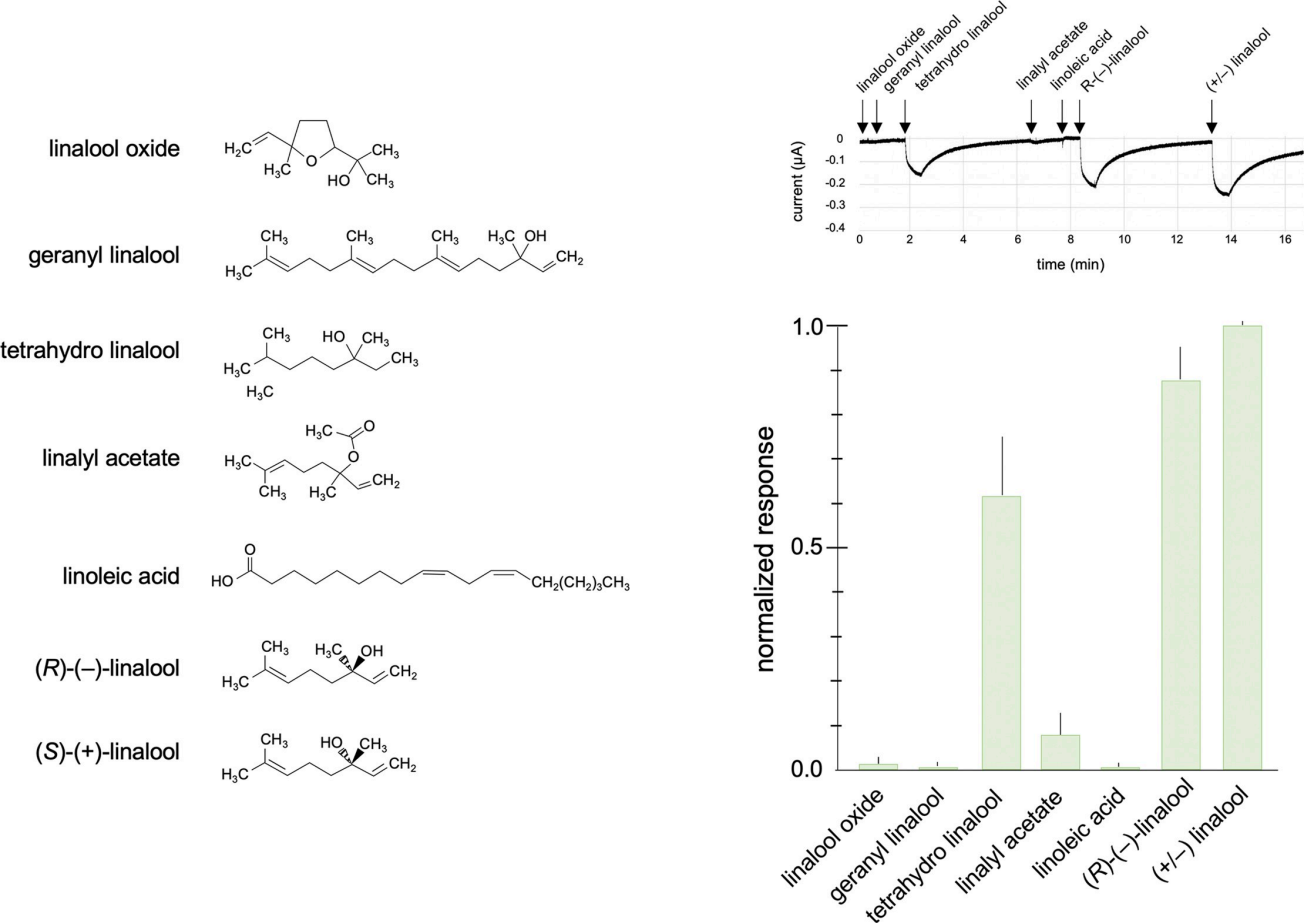
AgamOr29 is Tuned to Linalool. Normalized mean responses of AgamOr29 + AgamOrco oocytes (n = 10) to structurally-related linalool compounds. Standard errors are indicated in the positive direction only. Chemical structures are show to the left of the graph. A representative trace from an oocyte recording is shown above.

To characterize the specificity of AgamOr29, we tested six additional compounds with structural similarity to linalool against oocytes expressing the odorant receptor complex. The receptor showed peak amplitude responses to linalool that were approximately 10-fold and 2-fold greater than responses to linalyl acetate and tetrahydro linalool, respectively (Fig 6). Importantly, the response to an enantiomeric blend of (*R*)-(–)-linalool plus (*S*)-(+)-linalool produced a higher response than (*R*)-(–)-linalool alone (Fig 6). Although we sought to test the responsiveness of AgamOr29/Orco to pure (*S*)-(+)-linalool, the isomer was not commercially available and customized synthesis of the compound was prohibitively expensive.

### Concentration dependency of AgamOr29 linalool responses

**Fig 7:**
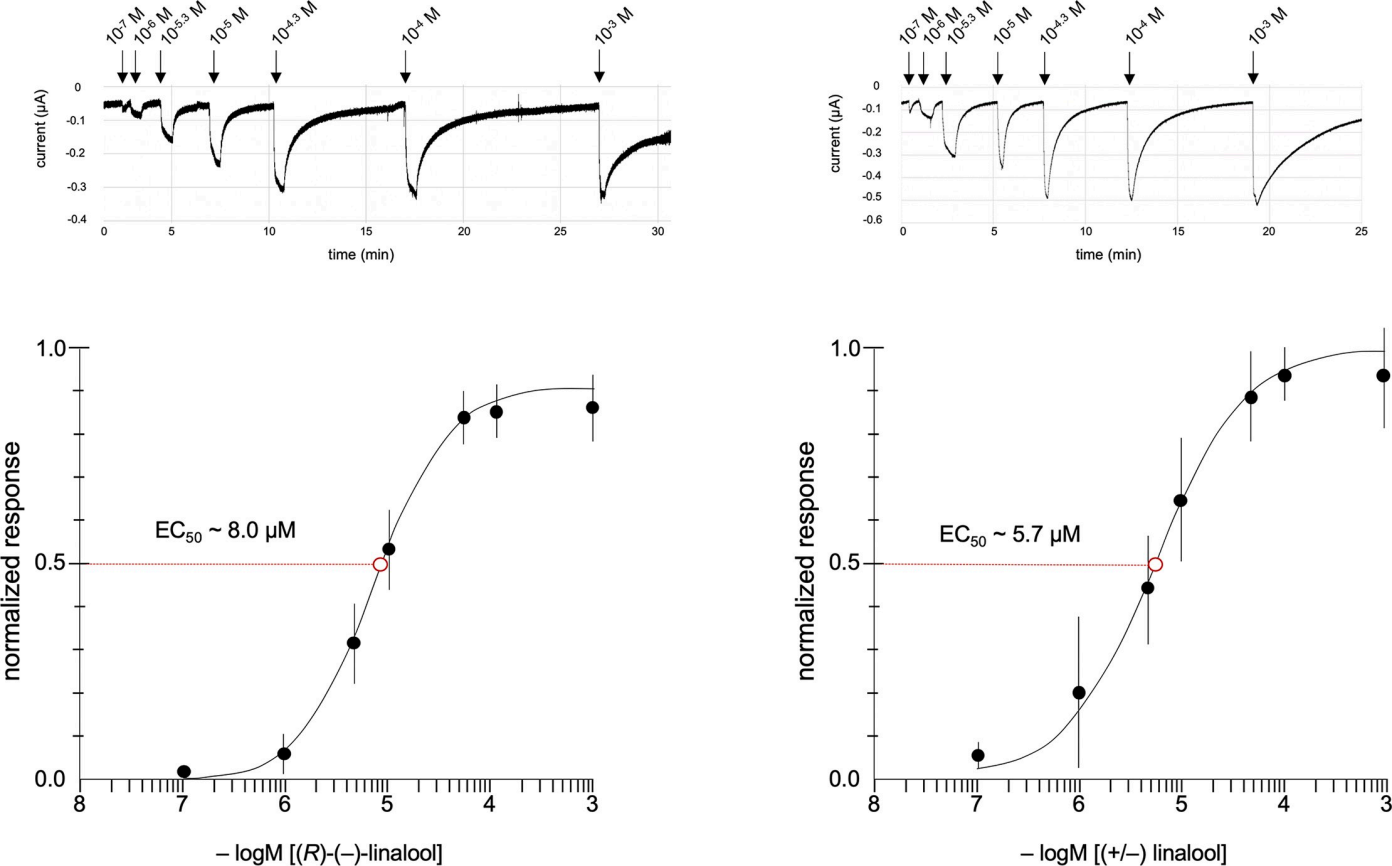
AgamOr29 concentration response curves. Normalized mean responses of AgamOr29 + AgamOrco oocytes (n = 12, n = 9) to (*R*)-(–)-linalool (left)and (+/–) linalool (right). Red dashed lines indicate estimated EC_50_ values of ~ 8.0μM and ~ 5.7μM, respectively. Representative traces from oocyte recordings are shown above each curve.

Another hallmark of receptor-ligand combinations is concentration dependency. In our experimental conditions, AgamOr29/Orco conferred concentration-dependent responses to oocytes that were subjected to dilutions of linalool from 10^−7^ M to 10^−3^ M (Fig 7). The resulting electrophysiological responses were fitted to sigmoid curves and half-maximal effective concentration values (EC_50_) values were calculated for isomers of linalool (Fig 7). (*S*)-(+)-linalool produced the lowest EC_50_ of ~ 5.7μM in comparison to the ~8.0μM EC_50_ value for (*R*)-(–)-linalool.

## Discussion

Linalool is an important compound to the chemical ecology of insects and has been shown to be a powerful modulator of insect behavioral traits. Interestingly, damaged maize seedlings (*Zea mays* var. Golden Queen) respond to herbivore attacks, with the production of linalool serving as an attractant to the natural enemies of herbivores, while inadvertently attracting additional *Spodoptera frugiperda* herbivorous larvae [44]. Many plants have been shown to be attractive to mosquitoes, which are equally attracted to plant extracts and to synthetic blends of the plant scent profiles [45–47]. Linalool is an interesting ligand due to its association with flowering plants and may therefore provide a chemical cue that helps to orient mosquitoes toward sources of nectar. AgamOr29 is most highly expressed in the labellar lobes of both sexes (Fig 3), which reinforces the hypothesis that this receptor is involved in short range chemical sensing and influences behavioral responses such as nectar feeding [40]. Linalool has also been identified as a component of human sweat and therefore may represent a multi-contextual odor cue that can act as a bloodmeal host attractant for Anophelines [48]. Low level expression of AgamOr29 in other head appendages may also influence behavioral responses.

AgamOr29 homologs encoded in the genomes of Anopheline species are likely to represent orthologous genes as they are extremely similar in length and amino acid identity, as well as having absolutely conserved intron positions (Fig 1). The phylogenetic relationships of Or29 orthologs across Anophelines agrees with previously defined species relationships, including the *A*. *gambiae* complex. The high degree of conservation of Or29 suggests that linalool perception plays an important role in the life histories of Anophelines. It will be interesting to compare the functionalities of Or29 orthologs across species and to conduct behavioral assays in *A*. *gambiae*, in response to linalool enantiomers, especially in an Or29 knockout. Moreover, the highly similar chemoreceptor, Or53, which is present in all available Anopheline genomes, supports an evolutionary history whereby Or29 is paralogous to Or53, having duplicated and diverged within the Cellia lineage (Fig 2). The conservation of these two receptors afford an interesting opportunity to study their functional significance across multiple vector species.

Although previous work has shown Anopheline odorant receptor responses to terpenes, our in-depth deorphanization of a mosquito terpene receptor is, to our knowledge, the first of its kind and is likely to represent an important aspect of the chemical ecology of Anophelines [4]. Here, we have shown that linalool selectively activates AgamOr29 in the low micromolar (between 5 and 10μM) concentration range consistent with the idea that it is the cognate odorant ligand for this receptor. It is important to note that the exact composition of the mixture of linalool used in our study is unknown, yet the presence of (*S*)-(+)-linalool in the mixture elicits an EC_50_ value almost half that of (*R*)-(–)-linalool. A previous study has suggested that spatial repellents of mosquitoes and biting midges are more effective at chiral linalool mixtures where (*S*)-(+)-linalool is at 65% or greater [49]. Nonetheless, the potency of (*S*)-(+)-linalool as an activator of Or29 remains to be experimentally determined. In addition, linalool has been found to be a component of human sweat and may be important to multiple aspects of the chemical ecology of mosquitoes, including bloodmeal host seeking [48]. Like many odors, linalool and enantiomeric compositions thereof, is likely to be perceived by mosquitoes in blends that elicit distinct valencies depending on blend composition and physiological state of the animal. Our tuning curve recordings also demonstrated that tetrahydro linalool elicited a moderate, but repeatable response from AgamOr29, likely due to its structural similarity to linalool. This supports an odor coding mechanism whereby chemical ligands occupy an external binding pocket on their cognate receptor(*s*) and that compounds with similar structure are able to occupy the orthosteric site, albeit with lower binding affinities than the natural ligand. This raises the possibility that non-natural chemical activators or inhibitors of Or29 could be identified that either interfere with or hyper-excite the receptor and provide a basis for push-pull vector control.

Several plant species that attract mosquitoes produce nectar and emit terpenoids including monoterpenes [50]. Terpenes have long been determined to be influential compounds emitted from plants and can be attractive or repulsive depending on the insect [51–54]. Terpene emission has also been shown to act as a defensive mechanism in conifers in response to bark beetles [55, 56]. Plant based volatiles associated with floral scents have been known to elicit significant orientation from both male and female mosquitoes. For example, *Culex pipiens* mosquitoes were shown to be attracted to floral extract of flowers from *Asclepias syriaca* and well as a synthetic floral-odor blend mimicking the floral extract [6]. Floral-odor blends such as the one described suggest their potential usage for monitoring or control of both male and female disease-vectoring mosquitoes.

Linalool is a plant-based volatile emitted from many flowers which suggests it may play a role in mosquito attraction to nectar sources and will be the subject of future studies. Furthermore, the strong conservation of Or29 and related paralogs in Anophelines provide targets for future studies of ligand-receptor interactions, especially to important plant-derived compounds like terpenes. With genetic engineering techniques available for creating knockout strains of mosquitoes it is feasible to generate Or29 null alleles that would be useful in determining the behavioral requirements of this receptor on linalool response. Moreover, Or29 provides a potentially useful target that could be used to specifically reduce biting by malaria-transmitting Anopheles mosquitoes via the development of novel excito-repellents or attractants for push-pull strategies that could be integrated into vector control programs. Such tools are becoming increasingly necessary as insecticide resistance erodes the effectiveness of national vector control programs worldwide [57–60].

## Supporting information

S1 Table Chemical compounds, CAS numbers, and suppliers used in this study. (XLSX)

S2 Table Identity matrix for Anopheline Or29 orthologs. Numbers indicate pairwise amino acid identities across all Anopheline Or29 orthologs. VectorBase peptide identifiers are provided in parentheses.
(XLSX)

S3 Table Raw data for *Xenopus laevis* oocyte recordings. Numbers indicate oocyte response amplitudes (μA).
(XLSX)

S1 File Conceptual translations of Anopheline Or29 orthologs. FASTA formatted amino acid sequences (single letter code).
(TXT)

S2 File Coding sequences for Anopheline Or29 orthologs. FASTA formatted DNA sequences.
(TXT)

S1 Fig Amino acid alignment of all Anopheline Or29 orthologs. **Single letter code for amino acids in alignment.** Identical amino acids (single letter code) are shaded gray, while similar amino acids are shown in bold type. Inverted triangles denote intron positions with numbers indicating intron frame.
(TIF)

S2 Fig Amino acid alignment of *An*. *gambiae* Or29 and *An*. *gambiae* Or53. Identical amino acids (single letter code) are shaded gray, while similar amino acids are shown in bold type. Inverted triangles denote intron positions with numbers indicating intron frame.
(TIF)
