# Supplementary figures and images for: An odorant receptor from Anopheles gambiae that demonstrates enantioselectivity to the plant volatile, linalool
Source: PLoS One. 2019 Nov 21;14(11):e0225637. doi: 10.1371/journal.pone.0225637 (PMC6872167; doi:10.1371/journal.pone.0225637)

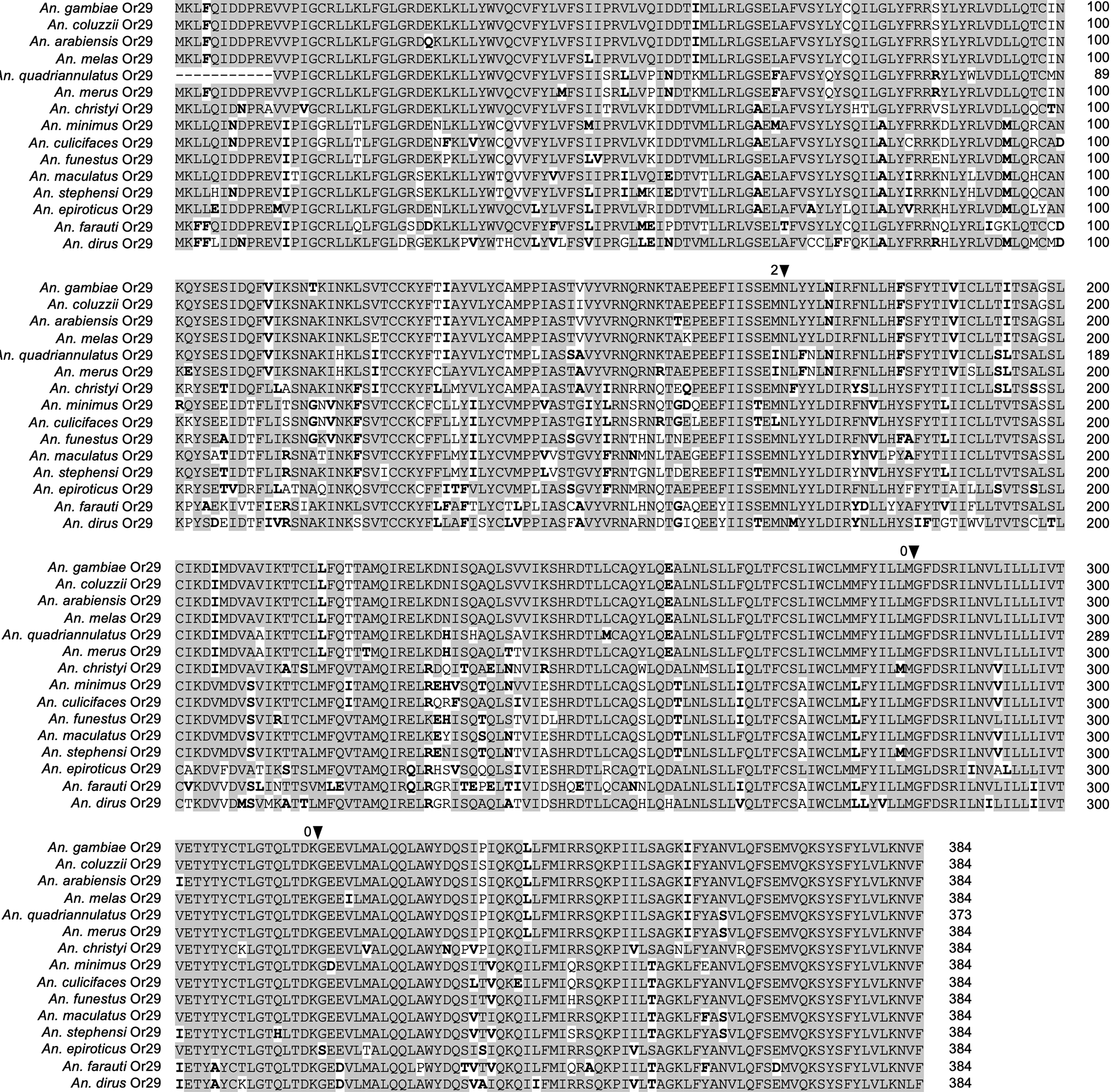

Supplement: S1 Fig — Single letter code for amino acids in alignment. Identical amino acids (single letter code) are shaded gray, while similar amino acids are shown in bold type. Inverted triangles denote intron positions with numbers indicating intron frame. (TIF) [file pone.0225637.s006.tif]

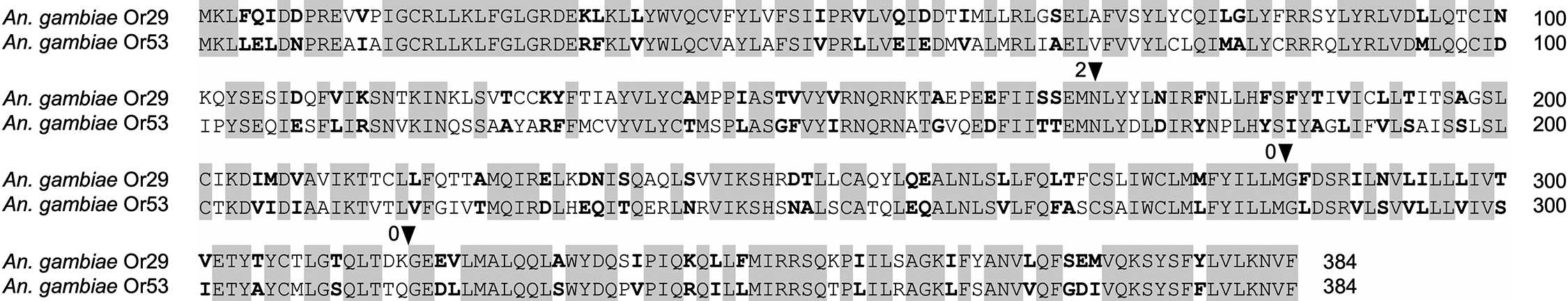

Supplement: S2 Fig — Identical amino acids (single letter code) are shaded gray, while similar amino acids are shown in bold type. Inverted triangles denote intron positions with numbers indicating intron frame. (TIF) [file pone.0225637.s007.tif]
